# Bipolar Tumor-Associated Macrophages in Ovarian Cancer as Targets for Therapy

**DOI:** 10.3390/cancers10100366

**Published:** 2018-09-29

**Authors:** Vijayalaxmi Gupta, Fiona Yull, Dineo Khabele

**Affiliations:** 1 Division of Gynecologic Oncology, Department of Obstetrics and Gynecology, University of Kansas Medical Center, Kansas City, KS 66160, USA; vgupta@kumc.edu; 2 Department of Pharmacology, Vanderbilt University, Nashville, TN 37232, USA; fiona.yull@vanderbilt.edu; 3 Vanderbilt-Ingram Cancer Center, Nashville, TN 37232, USA; 4 The University of Kansas Cancer Center, Kansas City, KS 66160, USA

**Keywords:** ovarian cancer, tumor-associated macrophages, peritoneal metastasis, tumor microenvironment

## Abstract

Ovarian cancer, a rare but fatal disease, has been a challenging area in the field of gynecological cancer. Ovarian cancer is characterized by peritoneal metastasis, which is facilitated by a cross-talk between tumor cells and other cells in the tumor microenvironment (TME). In epithelial ovarian cancer, tumor-associated macrophages (TAMs) constitute over 50% of cells in the peritoneal TME and malignant ascites, and are potential targets for therapy. Here, we review the bipolar nature of TAMs and the evolving strategies to target TAMs in ovarian cancer.

## 1. Introduction

Ovarian cancer is a rare but often fatal disease. Despite accounting for only 2.5% of all female cancers, ovarian cancer represents 5% of cancer deaths, and is the leading cause of gynecologic cancer death, in the United States [1,2]. The primary cause of death and the most common presence, in a high-grade serous epithelial ovarian cancer, is a peritoneal metastasis. Metastasis in epithelial ovarian cancer is characterized by ascites and tumor implants, that typically disseminate throughout the peritoneal cavity, along the lining of the peritoneum, the omentum, and the serosal surfaces of the viscera.

Peritoneal metastasis is regulated by cross-talk between tumor cells and the tumor microenvironment (TME). The TME is a dynamic cellular environment within an extracellular matrix surrounding the tumors, which contain a heterogeneous group of cells, including macrophages, lymphocytes, mesenchymal stem cells, fibroblasts, blood vessels, pericytes, and adipocytes [3,4]. Macrophages are converted into tumor-associated macrophages (TAMs), primarily through the release of cytokines, chemokines, and growth factors, secreted from tumor cells and other cells in the TME.

In epithelial ovarian cancer, TAMs constitute over 50% of cells in the peritoneal tumor implants and the ascites. TAMs are plastic and heterogeneous. Depending on the TME and the extracellular stimuli, macrophages exhibit two main phenotypes along a spectrum, the anti-tumorigenic (M1-like) and the pro-tumorigenic (M2-like). M2-like macrophages contribute to an immune suppressive TME and promote cross-talk between tumor cells and other cells leading to an enhanced tumor-cell growth, invasion, and metastasis [3,4]. This bipolar and plastic nature of the TAMs has the potential to be harnessed for therapeutic purposes. Indeed, the concept of re-educating M2-like macrophages to convert them into M1-like tumoricidal phenotypes was introduced as a therapeutic strategy, almost two decades ago [5]. Here, we review the bipolar nature of the TAMs and the evolving strategies to target TAMs in ovarian cancer.

## 2. Macrophages in Epithelial Ovarian Cancer

**Figure 1:**
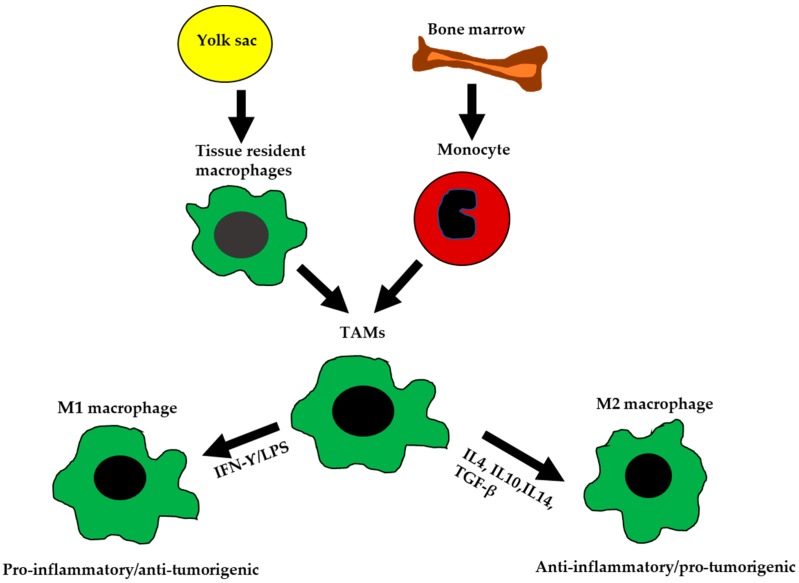
The ontogeny and polarization of M1 and M2 macrophages. Tissue-resident macrophages are mainly derived from yolk sac during development. Tumor-associated macrophages (TAMs) are derived from tissue-resident macrophages, or by differentiation of monocytes from the bone marrow. TAMs are polarized into M1-like or M2-like phenotypes based on signals received from the microenvironment (TME).

Macrophages are of myeloid lineage. They contribute to physiological homeostasis and constitute critical components of the innate immune response. Macrophages are involved in antigen presentation, phagocytosis, and other immuno-modulatory processes. Epithelial ovarian cancer TAMs originate from two main sources: (1) resident macrophages that arise from the embryonic yolk sac during development and (2) infiltrating macrophages that arise from the bone marrow monocytes (Figure 1) [6,7,8]. Both resident and infiltrating macrophages are heavily influenced by their cellular niche and transform into specific phenotypes, based on the signals they receive from the TME.

Resident macrophages are long-lived and maintained by local signals and the primary colony stimulating factor 1 (CSF-1), via the CSF-1 receptor (CSF-1R). Resident macrophages regulate immune responses and metabolic functions in a tissue-specific manner [9]. In ovarian cancer in mouse models, resident macrophages in the peritoneum are associated with GATA-6 [10]. In the omentum, one of the favored sites of the ovarian cancer peritoneal metastasis, resident macrophages are found in leukocyte-rich “milky spots” [11] and contribute to ovarian cancer cell invasion, both in the omentum and the rest of the peritoneal cavity [12,13]. In a mouse model of ovarian cancer, omental macrophages serve as a source of retinoic acid and other inducers to transport resident macrophages from the omentum to the peritoneum [10].

Infiltrating macrophages are short-lived and recruited from bone marrow monocytes. Infiltrating macrophages arrive in local tissue microenvironment and differentiate further into tissue-specific macrophages, which under homeostatic conditions, abide by the signals they receive from the surrounding microenvironment. In cancer, both the resident and the infiltrating macrophages in the TME, typically differentiate into pro-tumorigenic M2-like phenotypes.

Ascites is a hallmark of epithelial ovarian cancer, and its presence and volume are negatively related to prognosis [14]. TAMs, representing both the resident macrophages and the infiltrating macrophages, constitute a major fraction of the cells in epithelial ovarian cancer ascites [3,15,16]. TAMs in ovarian cancer ascites are primarily M2-like and pro-tumorigenic, with features similar to resident peritoneal macrophages, expressing genes involved in extracellular matrix remodeling, such as PCOLCE2 [17]. A sub-set of TAMS, found in ascites, are more similar to infiltrating macrophages. These are M1-like, expressing high levels of IFN-ƴ, which induces an IL-12-mediated cytotoxic response against tumor cells [18].

## 3. Bipolar Macrophages

A mixed population of TAMs exists in the TME of epithelial ovarian cancer [19]. Classically activated M1 and alternatively activated M2 are the two main phenotypes that represent a spectrum of functions (Figure 1 and Table 1) [20,21,22]. TAMs in the peritoneal cavity and ascites are primarily M2-like and are pro-tumorigenic. This polarization of TAMs towards M2 and M2/M1 ratios, has the potential use as a predictive and prognostic marker. For example, the ratio of M1/M2 is associated with an improved ovarian cancer prognosis [23]. In contrast, the ratio of CD163+ M2-like macrophages to the total CD68 macrophages (CD163/CD68) is a poor prognostic factor [24]. In addition, higher levels of CD163+ M2-like macrophages correlates with elevated IL6, and IL-10, and a shorter relapse-free survival [25].

### 3.1. M1 Macrophages

M1-like polarized macrophages are classically activated via the Th1 immune pathway. Th1 cells are mainly type 1 immune cells, which secrete cytokines, such as IFN-ƴ, IL-12, and TNF. They activate macrophages to induce inflammatory signaling pathways that exert tumoricidal effects [26]. Thus, the role of M1 macrophages is primarily pro-inflammatory, and they aid in killing pathogens and cancer cells.

M1 macrophages are critical for recruiting tumor-infiltrating lymphocytes that exhibit tumoricidal properties [27]. M1 macrophages secrete chemokines and cytokines to recruit T cells. Tumors with T cells have 14 times higher levels of macrophage-secreted chemokine mRNA levels, compared to tumors without T cells. Macrophage-derived chemokines delay the recurrence of ovarian cancer from 6 months to later than 40 months. Patients diagnosed with metastatic ovarian cancer, and tumors that contain tumor-infiltrating T cells, have a significantly improved clinical response to treatment, and a 38% overall five-year survival rate, as compared to a 4.5% survival rate in patients whose tumors had no T cells [27,28]. Tumor-infiltrating T cells recruited by M1-like macrophages induce tumors to express high levels of IFN-γ, IL-2, and other anti-tumorigenic cytokines. Tumors devoid of T cells have high levels of vascular endothelial growth factor (VEGF) expression, which contributes to the angiogenesis and a pro-tumorigenic TME. Glypican-3 (GPC3) enhances M1 macrophage recruitment and increases the secretion of IL-12 and TNF-alpha in ascites of GPC3 expressing mouse models of ovarian cancer [29]. Further, GPC3 is associated with an increased CD8+ T cell infiltration into the TME, induction of apoptosis of tumor cells, decreased ascites formation, and improved survival. Thus, M1 macrophage-derived chemokines, play a key role in recruiting cytotoxic T cells into the tumor microenvironment.

### 3.2. M2 Macrophages

M2-like macrophages are alternatively activated via the type 2 (Th2) immune pathway. Th2 immune cells secrete cytokines such as IL-4 and IL-5, that induce antibody formation. In general, M2 macrophages are anti-inflammatory and are involved in wound healing via tissue remodeling and the secretion of the extracellular matrix. In the setting of the TME in ovarian cancer, TAMs are primarily M2-like and are pro-tumorigenic. M2 TAMs support angiogenesis, tumor cell growth, migration, invasion, and metastasis [30,31]. This observation was supported by another group that showed that advanced ovarian cancers, with infiltration of M2 macrophages, are associated with poor survival [32].

M2 macrophages enhance cell proliferation in epithelial ovarian cancer cells via the MMP9/HB-EGF axis [33]. Sphere-forming ability is one of the hallmarks of cancer cells that are capable of metastasis. TAMs aid sphere formation and tumor growth, by secreting the epidermal growth factor (EGF) [34]. The EGF leads to integrin (αMβ2) upregulation, on TAMs, and an increased EGFR and ICAM-1 expression, on cancer cells. The elevated EGFR, in tumors, further activates the VEGF/VEGFR pathway in neighboring tumor cells, and thus supports cell proliferation and metastasis. M2-like macrophages facilitate the cell adhesion of ovarian cancer cells to mesothelial cells by causing the mesothelial cells to over-express P-selectin [35]. This mechanism likely supports the epithelial ovarian cancer spread, along the mesothelial-lined peritoneal cavity.

### 3.3. Molecular Mechanisms of Macrophage Polarization

The precise mechanism that regulates TAM polarization is an area of ongoing investigation. Although interferon regulatory factor IRF5 is the main transcription factor for M1 macrophages [36], an advanced transcriptome analysis unveiled additional transcription factors including, IRF3, Signal transducer, and activator of transcription (STAT) STAT1, STAT5. Hypoxia-inducible factor (HIF-1), nuclear factor kappa B (NF-κB) heterodimer, containing p65–p50, are major regulators of inflammatory chemokines and cytokines that polarize macrophages M1 phenotypes [37].

The main transcription factor for M2 polarization is IRF4 [38]. Proteomic analysis, comparing the proteins and transcripts of the resting and the M2 macrophages, revealed other transcription factors, such as STAT3 and STAT6, the NF-κB homodimer p50–p50, HIF-2, PI3K, AKT, and transglutaminase 2 (TGM2), which were associated with M2 polarization [39]. TGM2 is an enzyme with multiple functions, including cross-linking proteins, cell proliferation, and apoptosis [40]. Together, these transcription factors produce anti-inflammatory cytokines and chemokines typical of Th2 type immune cells.

MicroRNAs are also involved in macrophage polarization [41]. miR-216a is associated with M1 macrophage polarization, through telomerase activation, via the Smad3/NF-κB pathway [42]. Interestingly, miR-216a enhances p53 and p16 expression, which are suppressed in ovarian cancer. This suggests that increasing miR-216a through indirect means could be exploited therapeutically, in ovarian cancer. Reactive oxygen species (ROS) polarize macrophages to M1-like phenotypes [43,44]. HOXA9 polarizes peritoneal macrophages to M2-like phenotypes [45]. Thus, these molecular pathways offer additional means for therapeutically exploiting the bipolar nature of macrophages.

## 4. Inflammation and TAMs in Ovarian Cancer

Inflammation is one of the classic characteristics of cancer and is integral to cancer initiation, progression, and metastasis. Macrophages facilitate ovarian cancer peritoneal metastasis, via inflammatory pathways, mediated by cytokines and chemokines [46]. The NF-κB pathway provides an important link between inflammation and many types of cancer, including ovarian cancer [47].

Ascites derived from a syngeneic mouse model of ovarian cancer, contains macrophages as dominant cell populations [48]. Macrophage cell density increases proportionately to the volume of ascites and tumor progression. In this model, tumor cells at advanced stages have enhanced NF-κB activation. The peritoneal spread of cancer cells during tumor progression is associated with an increase in the number of M2 macrophages, but had a marginal effect on the number of M1 macrophages. Further, M2 macrophage levels are reduced by inhibiting NF-κB, in the tumors. The p50 component of NF-κB regulates M2-dependent inflammation and a lack of p50, leads to the elevated M1-associated inflammatory processes [49]. This provides an encouraging evidence that the ratio of M1/M2 macrophages can be shifted by targeting NF-κB.

Other factors linking inflammation and epithelial ovarian cancer, include serum amyloid A (SAA1/2) and macrophage migration inhibitory factors (MIF). Accumulation of serum amyloid A (SAA1/2) is associated with inflammation in epithelial ovarian cancer, via the TNF-alpha mediated activation of NF-κB [50]. Normal human ovarian tissues express little or no SAA1/2, whereas, ovarian cancers express high levels of SAA1/2 [43]. Elevated levels of MIF are found in ascites and in the circulation of ovarian cancer patients [51,52]. MIF levels correlate with the histological grade of the cancer tissue, disease prognosis, and platinum sensitivity [53]. MIF reduces natural-killer group 2, member D (NKG2D) expression, and prevents the natural killer (NK) cells from exerting their tumoricidal effects. NKG2D, under normal circumstances, activate the tumoricidal properties of NK and T cells. TME releases ligands for NKG2D and depletes NK cells, which in turn, increases the ratio of anti-tumorigenic CD163+ CD206+ M2-like macrophages in the TME. Soluble NKG2D ligands in ovarian cancer ascites indicated poor prognosis and decreased memory effector T cells [54].

## 5. TAMs as Therapeutic Targets

TAMs play a critical role in epithelial ovarian cancer tumorigenesis and, therefore, are promising targets for therapy. Evolving therapeutic approaches fall into three broad categories, that include strategies to (1) Block migration of monocytes to the TME; (2) re-polarize macrophages to increase the ratio of M1 to M2-like macrophages; and (3) inhibit immune-signaling pathways in macrophages.

### 5.1. Block Migration of Monocytes to the TME

Tumor cells and other cells in the TME release cytokines, chemokines, and growth factors that attract monocytes to the TME. This has been demonstrated, in vitro, using ovarian cancer cell lines, as well as, in vivo, using mouse models and some clinical settings.

#### 5.1.1. CSF-1 and CSF-1R

In clinical studies, CSF-1 and CSF-1R expression upregulation in epithelial ovarian cancer have been associated with poor prognosis [55]. The survival, proliferation, and differentiation of monocytes and macrophages are dependent on the CSF1R pathway [56]. In the syngeneic mouse model of ovarian cancer, GW2580, a selective CSF1R kinase inhibitor significantly reduces ascites fluid buildup and the infiltration of M2 TAMs [57]. Further, inhibiting the CSF-1R, partly overcomes anti-VEGF resistance [58], and the CSF-1R disruption results in macrophage depletion, which supports a direct role of the CSF-1R in macrophage recruitment [59]. Currently, active clinical trials targeting CSF1R on M2 macrophages, involve PLX3397, in combination with anti-PD-1 pembrolizumab (Clinical trial # NCT02452424), and Cabiralizumab (antibody against CSF1R), in combination with anti-PD-1 monoclonal antibody Nivolumab (NCT02526017). A clinical trial using LY3022855, a CSF1R inhibitor in combination with anti-PDl1 monoclonal antibody Durvalumab, or anti-cytotoxic T-lymphocyte associated protein 4 monoclonal antibody Tremelimumab, is currently recruiting (NCT02718911).

#### 5.1.2. CCL2

CCL2 is also known as MCP-1 (CC motif ligand 2 or macrophage chemoattractant protein-1), is a chemokine that plays a key role in monocyte recruitment to the TME. Epithelial ovarian cancer cells release CCL2/MCP-1 to attract monocytes and convert them to TAMs, within the TME [60]. A plant-derived product, 9-hydroxycanthin-6-one reduces the MCP-1 expression in ovarian cancer cells and inhibits macrophage recruitment [61]. Interestingly, using a mouse model, it was seen that CCL2/MCP-1 is crucial for Th2 immune responses. MCP-1^-/-^ mice do not induce the Th2 response and express low levels of IL-4, IL-5, and IL-10 [62]. Monocytes and macrophages express CCR2, which is a receptor for CCL2. Thus, the CCL2/CCR2 axis represents an attractive target for ovarian cancer therapy. A CCR2 antagonist RS504303 that is under development, significantly reduces bone-marrow derived monocyte cell migration, in mouse [63]. A clinical trial using an anti-CCl2 antibody, known as CNTO 888, in combination with gemcitabine or paclitaxel, and carboplatin or docetaxel, has been completed (NCT01204996).

#### 5.1.3. Drugs

Bisphosphonates deplete monocytes/macrophages in ovarian cancer. In a syngeneic mouse model of ovarian cancer, clodronate reduces TAMs by inhibiting cytokine secretion, which decreases angiogenesis [64]. In patients with epithelial ovarian cancer, transient depletion of peritoneal macrophages using liposomal alendronic acid potentiates an adoptive immunotherapy [65].

Trabectedin, a marine-derived anti-tumor compound, depletes macrophages in mouse models [66]. A phase 2 clinical trial of trabectedin in ovarian cancer patients, showed a significant depletion of blood monocytes, as well as a reduction in CCL2 levels, in TAMs and ovarian tumor cells [67]. However, trabectedin as a single agent has limited efficacy. An alternate strategy to deplete TAMs is to exploit elevated expression levels of folate receptor-2 (FOLR2) that has been found in human and murine ovarian cancer TAMs, and use G-5 methotrexate nanoparticles to target these TAMs [68].

### 5.2. Re-Polarize Macrophages to Increase the Ratio of M1 to M2-Like Macrophages

Notch signaling plays a crucial role in M1 polarization in a mouse model, where macrophages with an active Notch display anti-tumor properties. Most of the following studies, unless otherwise stated, were carried out using a mouse model. When Notch signaling is blocked, M2 macrophages are polarized and resist M1 activators [69]. CCL2, apart from recruiting monocytes, enhances M2 polarization as well [70]. Activation of the peroxisome proliferator-activated receptor ƴ (PPARƴ)/NF-κB axis, in ovarian cancer stem cells, induces M2 polarization [71].

An unexpected observation made by our group revealed that inhibition of NF-κB in a syngeneic mouse model of ovarian cancer, increased pro-tumorigenic M2 macrophages, which promoted ascites, an increased expression of pro-tumorigenic soluble factors (such as VEGF in ascites fluid), and an infiltration of more M2 macrophages into the TME [48,72]. These results suggest that the activation of NF-κB in TAMs, not tumor cells, could be a viable therapeutic strategy. Indeed, NF-κB transfected TAMs, display anti-tumorigenic properties in mice harboring solid tumors, which on treatment showed elevated M1 phenotype favoring Th1 cytokines and reduced Th2 cytokines [73].

Antibiotics and natural products modulate macrophages. Doxycycline is a common antibiotic that reduces pro-angiogenic properties of M2 macrophages, in neovascular age-related macular degeneration models [74]. Among natural products, deoxyschizandrin, a phytochemical extracted from berries, significantly reduces the pro-tumorigenic activity of TAMs by inhibiting M2 macrophages [75]. In addition to blocking macrophage recruitment to tumor sites, 9-hydroxycanthin-6-one, inhibits M2 polarization in ovarian cancer [61]. Neferine, another plant-derived product, was found to inhibit M2-macrophages in an OVHM xenograft mouse model [76].

### 5.3. Inhibit Immune Signaling Pathways in Macrophages

The tumor-associated PD-L1 expression has been investigated by several investigators [77,78]. In addition, macrophages associated with primary and metastatic high-grade serous, ovarian cancer express PD-L1 [77]. A comparison of the TME of primary and recurrent epithelial ovarian cancer showed interesting trends, regarding the effect of T cells and macrophages, on survival. Recurrent tumor TME, with higher immune cell recruitment and higher TAMs, have better survival [79]. In clinical studies, expression of PD-L1, by both immune cells and tumor cells in recurrent tumors, leads to an active immune response and imparts better survival in recurrent cancer, as compared to primary cancer, where only the immune cells express PD-L1.

They further explained that the phenotype of regulatory T cells (Tregs), in primary and recurrent cancer, is different, with recurrent cancer expressing more CD25+ Tregs, which are indicators of better prognosis. Thus, they concluded that the dynamics between TAM PD-L1 expression and cytotoxic vs. Tregs create an imbalance that favors survival. PD-L1 expression was significantly higher in ovarian cancer than in other cancers and coincided with poor prognosis [78]. Although PD-L2 expression was associated with poor prognosis, there was no significant difference in PD-L2 between primary and recurrent ovarian cancer. Their most interesting finding was that the tumor cell PD-L1 expression was inversely proportional to the CD8 expression of intraepithelial tumor-infiltrating lymphocytes (TILs). Further, their findings supported CD8+ TILs as a positive predictor of overall survival and progression-free survival, in ovarian cancer. B7-H4 protein and mRNA is highly expressed in ovarian cancers and is involved in epithelial cell transformation [80]. B7-H4 inhibits T cell activation, thereby, halting host anti-tumor response, leading to a tumor escape from immune surveillance. Earlier reports from this group showed that B7-H4 is also expressed in ovarian tumor-associated macrophages, and similar to tumor B7-H4, these macrophages also suppress tumor immunity [81]. When normal blood monocytes were incubated with tumor ascites, elevated levels of B7-H4 was observed, whereas, the serum-free medium showed no such effect, thereby suggesting that B7-H4 expression is regulated by the tumor microenvironment, specifically IL-6 and IL-7.

**Figure 2:**
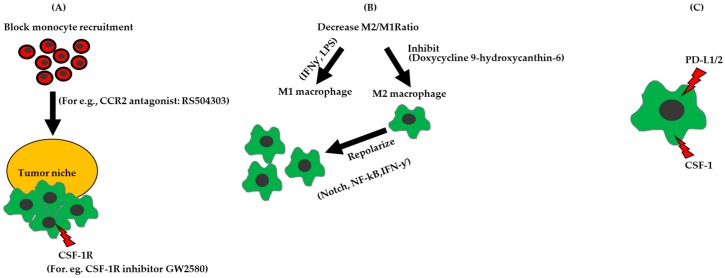
Strategies for targeting TAMs in ovarian cancer. (**A**) Block monocyte recruitment to the tumor niche. (**B**) Chemical intervention to increase M1/M2 ratio by inhibiting M2 polarization, increasing M1 polarization by using Interferon gamma (IFN-ƴ, Lipopolysaccharide (LPS)) or by repolarizing M2 to M1 by adding IFN-ƴ or regulating the Notch, NF-κB. (**C**) Inhibit immune signaling pathways on macrophages, for e.g., CSF-1, VEGFR, which promotes angiogenesis, and PD-L1, which inhibits T cell activity.

Current clinical trials that target PDL1/2 and PD1/2 axis include patients diagnosed with ovarian cancer. A clinical trial for platinum-resistant ovarian cancers involves a combination of the anti-PD-L1 antibody Atezoliuzub with Bevacizumab (NCT02659384). Another clinical trial, for advanced ovarian tumors and recurrent ovarian cancer is investigating a combination of an anti-PDL1 antibody MEDI4736 with Olaparib and/or Cedinarib (NCT024844004). Designing strategies to alleviate immune suppression, by reducing monocyte recruitment, decreasing the M2/M1 ratio, and targeting TAMs in combination with with immune checkpoint inhibitors, could represent attractive targets that switch the innate immunity balance in favor of tumor cell death (Figure 2).

## 6. Conclusions

In epithelial ovarian cancer, TAMs mediate progressive ovarian cancer and thus present an attractive target to develop anti-cancer regimens, as they are involved in all stages of ovarian cancer development. M1 macrophages, on the other hand, represent anti-tumorigenic TAMs. An advantage of using TAMs as anti-cancer targets is their genomic stability, which could provide a means of alleviating drug resistance. A deeper understanding of mechanisms behind macrophage polarization, will aid in developing strategies to enhance M1 macrophage polarization or shift the balance between M1 and M2 towards anti-tumorigenic M1 macrophage population. TAMs represent a plastic immune cell population amenable to manipulation and re-education and repolarizing M2 to M1 tumoricidal phenotypes. The affinity of TAMs to the peritoneal TME and the ascites in epithelial ovarian cancer, offers a future potential for targeted intraperitoneal treatment, in combination with chemotherapy drugs.

Despite their promise, clinical implementation of macrophage-based therapies has been limited. The main challenges in targeting TAMs, are their complexity and heterogeneity in the context of the TME and the likely need to combine macrophage-based therapies with other anti-tumor agents. Cross-talk between tumor cells and other cells in the TME is complex. Ongoing research to ‘deconvolute’ elements of the TME will lead to a better understanding of how to strategically target dominant cell populations, such as macrophages in different TME niches [4]. Classic definitions of M1 and M2 do not fully encompass the full spectrum of macrophage function. Next generation single cell sequencing and flow methods will be required to better understand the most important functions of the sub-types of TAMs, in ovarian cancer.

## Figures and Tables

**Table 1:** Comparison of characteristics of M1 and M2 macrophages. Adapted from Krishnan, 2018 [19], Mantovani, 2002 [21], and Mantovani, 2004 [22].

Characteristics	M1 Macrophage	M2 Macrophage
Activation pathway	Th1 (Classical)	Th2 (Alternative)
Tumor relation	Anti-tumorigenic	Pro-tumorigenic
Inducers	LPS, IFN-gamma, IL-12	IL4, IL10, IL13, TGF-β, CCL2, CXCL4
Chemokines	CXCL9, CXCL10, CCL4, CCL10, CCL11	CCL17, CCL22, CCL24
Markers	CD86, CD80, iNOS, TLR2, TLR4, IL-1R, MHC-II	CD163, CD206, CCl18, IL-1RII, TGM2
Antigen processing/presentation	Yes–Increased MHCII, STAT1, NO production	No-Decreased MHCII, STAT-1, NO production
Function	Pro-inflammatory/Tissue damage/Pathogenic clearance/Anti-angiogenic	Anti-inflammatory/Tissue repair and remodeling/Fibrosis/Pro-angiogenic

Abbreviations: Major histocompatibility complex (MHC); signal transducer and activator or transcription 1 (STAT1); Nitric oxide (NO).
